
JOURNAL INFORMATION
==============================
NLM Title Abbreviation: ScientificWorldJournal
Journal ID: ScientificWorldJournal
NLM Journal ID: 101131163
Journal ID: TSWJ

The Scientific World Journal

ISSN: 2356-6140
EISSN: 1537-744X
Publisher: Wiley

ARTICLE INFORMATION
==============================
PMCID: PMC6084416
PMID: 30147558
DOI: 10.1100/tsw.2001.115
Article ID: 737341
Article version: 1
Subjects: Short Report

Lipotoxic Nephropathy Is More Severe in Male than Female Rats N with Non-Insulin Diabetes Mellitus (NIDDM)

Dominguez Jesus H. *
Xu Wei
Hawes John W.
Peterson Richard G.
Department of Medicine, Biochemistry, and Anatomy: IUMC and VAMC, Indianapolis, IN, 46202, USA
*Jesus H. Dominguez: jhdoming@iupui.edu
Publication date: 2001
Electronic publication date: 2001 Feb 9
Volume: 1
First page: 86
Last page: 86
Copyright: Copyright © 2001 Jesus H. Dominguez et al.
Copyright year: 2001
License: This is an open access article distributed under the Creative Commons Attribution License, which permits unrestricted use, distribution, and reproduction in any medium, provided the original work is properly cited.
License URL: https://creativecommons.org/licenses/by/3.0/

==============================
Miami *Nature Biotechnology* Short Reports
*TheScientificWorld* (2001) 1(S3), 86SR
ISSN 1532-2246; DOI 10.1100/TSW.2001.115

### LIPOTOXIC NEPHROPATHY IS MORE SEVERE IN MALE THAN FEMALE RATS N WITH NON-INSULIN DIABETES MELLITUS (NIDDM)

Jesus H. Dominguez*, Wei Xu, John W. Hawes , and Richard G. Peterson

Department of Medicine, Biochemistry, and Anatomy: IUMC and VAMC, Indianapolis, IN, 46202, USA
* jhdoming@iupui.edu

**INTRODUCTION.** Dyslipidemia is a prominent risk factor for nephropathy in NIDDM; as indicated by longitudinal studies (1,2). This association is not surprising, as the most severe examples of hypercholesterolemia are either familial, or caused by NIDDM (3). Moreover, dyslipidemia in NIDDM is also coupled with markedly increased levels of oxidized LDL (oxLDL) (4), and heightened oxidative stress is recognized to complicate NIDDM (5). However, specific renal toxicity of lipid oxidant (lipotoxicity) is not proven in NIDDM, perhaps because such proof is difficult to obtain in human investigations. We tested the hypothesis that lipotoxicity enhances the level of glomerular oxidized LDL receptor 1 (OLR1) in obese males rats with NIDDM, augmenting oxidant stress and resulting fibrosis.

**METHODS.** First generation ($F_1$) hybrid rats derived from the Zucker fatty diabetic strain (ZDF) and the spontaneous hypertensive heart failure rat (SHHF/Gmi-fa) were studied for 41 weeks on two diets. Three groups of rats, 10 obese males (OM5008), 13 obese females (OF5008), and 10 lean males (LM5008), were fed diet # 5008 (27 % protein, 17 % animal fat, 56 % carbohydrate, and 11.1 uMoles/gm lipid hydroperoxide). Two other groups, 3 OM12470 and 14 OF12470, were aging controls, and ate diet #12470 (10 % protein, 48 % animal fat, 42 % carbohydrate, and 4.5 uMoles/gm lipid hydroperoxide).

**RESULTS.** OM5008 rats had an atherogenic plasma lipid profile, with markedly elevated plasma cholesterol and hydroperoxides. Their glomeruli expressed OLR1 (antibody from Dr. M. Nagase) and accumulated 4-OH-nonenal (4HNE); the highly cytotoxic lipid peroxide end-product with mitochondria localization. OM5008 also had the highest levels of creatinine, renal Transforming Growth Factor β-1 (TGF β-1) and glomerulosclerosis. Remarkably, hypertension and hyperglycemia were not directly linked to renal injury.

| GROUP | Cholesterol (mM) | TBARS (uM) | Glomerular OLR1(*) | Renal 4-HNE (**) | Plasma Creatinine (uM) | Renal TGFβ1 Pg/mg prot |
|---|---|---|---|---|---|---|
| **OM5008 (#)** | 18.8 ± 2.9 | 22 ± 4 | 50 ± 5 | 2.43 ± 0.31 | 100 ± 16 | 715 ± 65 |
| **OF5008** | 7.7 ± 1.2 | 12 ± 1 | 10 ± 3 | 0.58 ± 0.15 | 46 ± 1 | 290 ± 72 |
| **LM5008** | 2.2 ± 0.1 | 8 ± 1 | 6 ± 2 | 0.75 ± 0.06, | 42 ± 1 | 55 ± 13 |
| **OM12470** | 9.9 ± 1.4 | 13 ± 1 | 7 ± 3 | 1.31 ± 0.12 | 50 ± 3 | 370 ± 46 |
| **0F12470** | 2.5 ± 0.2 | 10 ± 1 | 7 ± 2 | 0.51 ± 0.04 | 41 ± 1 | 272 ± 53 |

(*)Fraction of glomeruli expressing OLR1 ; (**) = Fraction of glomeruli loaded with 4-HNE, 4 = 75-100%, 3 = 50-75 %, 2 = 25 – 50 %, 1 = 0 – 25 % ;(#) significantly higher for all parameters, $p < 0.05$.

**CONCLUSIONS.** Glomerular expression of OLR1 is linked to deposits of 4HNE and glomerulosclerosis. We presume that in OM5008, OLR1 enhances glomerular uptake of oxidized LDL, causing greater oxidant stress, TGF β1 production, and glomerulosclerosis. In OF5008, renal protection from oxidants is conferred by blunted glomerular OLR1 expression and an effective renal antioxidant system.

**REFERENCES.**

1. 1. Ravid, M., Brosh, D., Ravid-Safran, D., Levy, Z., and Rachmani, R. (1998) Arch. Intern. Med. 11, 998-1004
2. 2. Yokoyama, H., Okudaira, M., Otani, T., Watanabe, C., Takaike, H., Miuira, J., Yamada, H., Mutou, K., Satou, A., Uchigata, Y., and Iwamoto, Y. (1998) Diabetes Care 21, 1080-1085
3. 3. Knopp, R.H. (1999) N. Engl. J. Med. 341, 498-511
4. 4. Yoshida, H., Ishikawa, T., and Nakamura, H. (1997) Arterioscler. Thromb. Vasc. Biol. 17, 1438-1446
5. 5. Delmas-Beauvieux, M.C., Peuchant, E., Thomas, M.J., Dubourg, L., Pinto, A.P., Clerc, M., and Gin, H. (1998) Clin. Biochem. 31, 221-228
